# Supplementary material for: Contributing to evidence-based veterinary medicine: A qualitative study of veterinary professionals’ views and experiences of client-owned companion animal research
Source: PLoS One. 2025 May 9;20(5):e0322902. doi: 10.1371/journal.pone.0322902 (PMC12063805; doi:10.1371/journal.pone.0322902)
Supplement: S1 Appendix — Script used during interviews with veterinary professionals in the study. (DOCX) [file pone.0322902.s001.docx]

**INTERVIEW SCHEDULE**

Outline of semi-structured interviews

Below are the prompts for the semi-structured interviews (individuals / pairs) and focus group discussions. Questions may be skipped or changed according to the flow of the interview and topics discussed by the participants. Participants will be encouraged to share experiences and stories they feel are relevant to the study.

*Introduction*

**Individual / pairs of interviews (veterinary professionals)**

Thank-you for taking the time to speak to me. This discussion should take around 1 hour but may be more or less depending on how much you want to say. The discussion will be recorded to assist with analysis. During our discussion I may take some notes but please do not feel discouraged and please continue talking. There are no right or wrong answers and any views you provide will be anonymised so that you cannot be identified. Are you happy with the information we have provided you (including the information sheet) and do you have any questions? *Check consent sheet returned – alternatively to be read out and participant to confirm verbal acceptance.*

*Prompts*

Icebreaker

- What area of the profession do you work in?
- Where did you train?
- How long have you been working as a veterinary surgeon / vet nurse?

Veterinary research – general

- Have you been involved in any research studies either as a student or following qualification?
- Has your hospital / practice been involved in any research studies?
- Have you done any formal post qualification training?
- How often do you read veterinary research papers?
- Do you use results of veterinary research studies to guide discussions with your colleagues / owners?
- How do you feel about practice-based veterinary research? What would make you want to be involved? What would make you reluctant to / or refuse to participate in a research study?
- How do you think owners feel about such studies? What information do you think they would want?

Patient-based veterinary interventional studies (i.e. where there are two groups / randomised / blinded)

** well designed studies with appropriate regulatory & ethical approvals*

- Have you any experience of human interventional studies e.g. clinical trials involving you or a relative / friend?
- How do you feel about interventional veterinary studies such as clinical trials? What would make you want to be involved? What would make you reluctant to / or refuse to participate?
- How do you think your practice / hospital clients would feel about these types of studies?
- Would you feel comfortable about approaching them to participate in such a study* if their animal was eligible?
- What would make you reluctant to ask an owner about participating in a study?
- What might make this type of conversation easier for you?
- What do you think owners would consider to be important?
