# Supplementary material for: Contributing to evidence-based veterinary medicine: A qualitative study of veterinary professionals’ views and experiences of client-owned companion animal research
Source: PLoS One. 2025 May 9;20(5):e0322902. doi: 10.1371/journal.pone.0322902 (PMC12063805; doi:10.1371/journal.pone.0322902)
Supplement: Attachment — Submitted filename: PONE-D-24-57716 (1). EBVM.pdf [file pone.0322902.s002.pdf]

PLOS ONE

Contributing to evidence-based veterinary medicine: a qualitative study of veterinary professionals' views and experiences of client-owned companion animal research

--Manuscript Draft--

**Manuscript Number:**	PONE-D-24-57716
**Article Type:**	Research Article
**Full Title:**	Contributing to evidence-based veterinary medicine: a qualitative study of veterinary professionals' views and experiences of client-owned companion animal research
**Short Title:**	Veterinary professionals' views on client-owned companion animal research
**Corresponding Author:**	Debra Catherine Archer, BVMS PhD<br>University of Liverpool<br>Neston, Wirral UNITED KINGDOM OF GREAT BRITAIN AND NORTHERN IRELAND
**Keywords:**	Evidence-based veterinary medicine; clinical veterinary research; Clinical Trials; animal research; veterinary professionals
**Abstract:**	Background: Research on the outcomes of veterinary treatments in dogs, cats and horses has important benefits for these animals and their owners. However, this information is not always available and the evidence base is often lower-quality than in human medicine. To identify ways to improve the generation of evidence, we investigated the views of veterinary professionals about research involving companion animal patients and their owners.<br>Methods and Findings: This qualitative study used semi-structured interviews with veterinary surgeons and registered veterinary nurses working in UK companion animal clinical practice. Interviews were conducted with 20 veterinary professionals from different clinical settings including both those with and without previous experience of research involving animals. Analyses revealed that veterinary professionals valued evidence-based information to help them make informed decisions about treatment with owners. However, there was often not enough available evidence. Veterinary professionals were willing to help produce this research evidence. However, lack of time and resources were key challenges and in addition, they did not always feel they had the necessary research skills, experience and support. Some participants also found it difficult to discuss participation in research with owners of their veterinary patients. They also had concerns about the amount and type of extra information they would need to give to owners. Veterinary professionals also faced a dilemma as their key role is to protect the welfare of animals that they treat, yet felt that there was the potential for some animals assigned to receive a specific treatment as part of a research study to be disadvantaged.<br>Conclusions: Companion animal research has important benefits for veterinary patients, their owners and for veterinary professionals. More funding, improved research training, resources, support networks and changes to current regulations are needed. Improved evidence would assist veterinary professionals and owners when making informed decisions around veterinary care.
**Order of Authors:**	Tamzin Furtado, PhD
	Elizabeth Perkins
	Debra Catherine Archer, BVMS PhD
**Additional Information:**	
**Question**	**Response**
**Financial Disclosure**<br><br>Enter a financial disclosure statement that describes the sources of funding for the work included in this submission. Review the submission guidelines for detailed	Yes

Powered by Editorial Manager® and ProduXion Manager® from Aries Systems Corporation

requirements. View published research articles from *PLOS ONE* for specific examples.<br><br>This statement is required for submission and **will appear in the published article** if the submission is accepted. Please make sure it is accurate.<br><br>**Funded studies**<br>Enter a statement with the following details:<br>• Initials of the authors who received each award<br>• Grant numbers awarded to each author<br>• The full name of each funder<br>• URL of each funder website<br>• Did the sponsors or funders play any role in the study design, data collection and analysis, decision to publish, or preparation of the manuscript?<br><br>Did you receive funding for this work?	
Please add funding details.<br>as follow-up to "**Financial Disclosure**<br><br>Enter a financial disclosure statement that describes the sources of funding for the work included in this submission. Review the submission guidelines for detailed requirements. View published research articles from *PLOS ONE* for specific examples.<br><br>This statement is required for submission and **will appear in the published article** if the submission is accepted. Please make sure it is accurate.	This work was funded by the University of Liverpool Policy Support Fund, which is funded by Research England. The funders played no part in the study design, data collection and analysis, decision to publish, or preparation of the manuscript.

Powered by Editorial Manager® and ProduXion Manager® from Aries Systems Corporation

**Funded studies**<br>Enter a statement with the following details:<br>• Initials of the authors who received each award<br>• Grant numbers awarded to each author<br>• The full name of each funder<br>• URL of each funder website<br>• Did the sponsors or funders play any role in the study design, data collection and analysis, decision to publish, or preparation of the manuscript?<br><br>Did you receive funding for this work?"	
Please select the country of your main research funder (please select carefully as in some cases this is used in fee calculation).<br>as follow-up to "**Financial Disclosure**<br><br>Enter a financial disclosure statement that describes the sources of funding for the work included in this submission. Review the submission guidelines for detailed requirements. View published research articles from *PLOS ONE* for specific examples.<br><br>This statement is required for submission and **will appear in the published article** if the submission is accepted. Please make sure it is accurate.<br><br>**Funded studies**<br>Enter a statement with the following details:<br>• Initials of the authors who received each award<br>• Grant numbers awarded to each author<br>• The full name of each funder<br>• URL of each funder website<br>• Did the sponsors or funders play any role in the study design, data collection and analysis, decision to publish, or preparation of the manuscript?<br><br>Did you receive funding for this work?"	UNITED KINGDOM - GB
**Competing Interests**	The authors have declared that no competing interests exist

Use the instructions below to enter a competing interest statement for this submission. On behalf of all authors, disclose any competing interests that could be perceived to bias this work—acknowledging all financial support and any other relevant financial or non-financial competing interests.<br><br>This statement is required for submission and **will appear in the published article** if the submission is accepted. Please make sure it is accurate and that any funding sources listed in your Funding Information later in the submission form are also declared in your Financial Disclosure statement.<br><br>View published research articles from *PLOS ONE* for specific examples.<br><br>**NO authors have competing interests**<br><br>Enter: *The authors have declared that no competing interests exist.*<br><br>**Authors with competing interests**<br><br>Enter competing interest details beginning with this statement:<br><br>*I have read the journal's policy and the authors of this manuscript have the following competing interests: [insert competing interests here]*<br><br>* typeset	
**Ethics Statement**<br><br>Enter an ethics statement for this submission. This statement is required if the study involved:<br><br>• Human participants<br>• Human specimens or tissue<br>• Vertebrate animals or cephalopods<br>• Vertebrate embryos or tissues	The study was approved by the University of Liverpool Veterinary Ethics Committee, number VREC 1193. All participants gave written or audio-recorded informed consent prior to participating.

Powered by Editorial Manager® and ProduXion Manager® from Aries Systems Corporation

• Field research<br><br>Write "N/A" if the submission does not require an ethics statement.<br><br>General guidance is provided below. Consult the submission guidelines for detailed instructions. **Make sure that all information entered here is included in the Methods section of the manuscript.**	

Powered by Editorial Manager® and ProduXion Manager® from Aries Systems Corporation

**Format for specific study types**<br><br>**Human Subject Research (involving human participants and/or tissue)**<br>• Give the name of the institutional review board or ethics committee that approved the study<br>• Include the approval number and/or a statement indicating approval of this research<br>• Indicate the form of consent obtained (written/oral) or the reason that consent was not obtained (e.g. the data were analyzed anonymously)<br><br>**Animal Research (involving vertebrate animals, embryos or tissues)**<br>• Provide the name of the Institutional Animal Care and Use Committee (IACUC) or other relevant ethics board that reviewed the study protocol, and indicate whether they approved this research or granted a formal waiver of ethical approval<br>• Include an approval number if one was obtained<br>• If the study involved *non-human primates*, add *additional details* about animal welfare and steps taken to ameliorate suffering<br>• If anesthesia, euthanasia, or any kind of animal sacrifice is part of the study, include briefly which substances and/or methods were applied<br><br>**Field Research**<br><br>Include the following details if this study involves the collection of plant, animal, or other materials from a natural setting:<br>• Field permit number<br>• Name of the institution or relevant body that granted permission	
**Data Availability**<br><br>Authors are required to make all data underlying the findings described fully available, without restriction, and from the time of publication. PLOS allows rare exceptions to address legal and ethical concerns. See the PLOS Data Policy and FAQ for detailed information.	No - some restrictions will apply

A Data Availability Statement describing where the data can be found is required at submission. Your answers to this question constitute the Data Availability Statement and **will be published in the article**, if accepted.<br><br>**Important:** Stating 'data available on request from the author' is not sufficient. If your data are only available upon request, select 'No' for the first question and explain your exceptional situation in the text box.<br><br>Do the authors confirm that all data underlying the findings described in their manuscript are fully available without restriction?	
**Describe where the data may be found in full sentences. If you are copying our sample text, replace any instances of XXX with the appropriate details.**<br><br>• If the data are **held or will be held in a public repository**, include URLs, accession numbers or DOIs. If this information will only be available after acceptance, indicate this by ticking the box below. For example: *All XXX files are available from the XXX database (accession number(s) XXX, XXX.*).<br>• If the data are all contained **within the manuscript and/or Supporting Information files**, enter the following: *All relevant data are within the manuscript and its Supporting Information files.*<br>• If neither of these applies but you are able to provide **details of access elsewhere**, with or without limitations, please do so. For example:<br><br>*Data cannot be shared publicly because of [XXX]. Data are available from the XXX Institutional Data Access / Ethics Committee (contact via XXX) for researchers who meet the criteria for access to confidential data.*<br><br>*The data underlying the results presented in the study are available from (include the name of the third party*	Data cannot be shared publicly due to issues of anonymity from the relatively small pool of respondents. Data are available via University of Liverpool Veterinary Ethics Committee (ivesethics@liverpool.ac.uk) for researchers who meet the criteria for access to confidential data.

and contact information or URL).<br>• This text is appropriate if the data are owned by a third party and authors do not have permission to share the data.<br><br>* typeset	
Additional data availability information:	

Main manuscript

Click here to access/download;Manuscript;Submission for review 02 Jan 2025.docx

Contributing to evidence-based veterinary medicine: a qualitative study of veterinary professionals' views and experiences of client-owned companion animal research

Short title: veterinary professionals' views on client-owned companion animal research

Key words: Evidence-based veterinary medicine; clinical veterinary research; clinical trials; animal research; veterinary professionals

Tamzin Furtado[1], Elizabeth Perkins [2], Debra C. Archer [1]

1.School of Veterinary Science, Institute of Infection, Veterinary and Ecological Sciences, University of Liverpool, Liverpool, United Kingdom. 2. Department of Primary Care and Mental Health, Institute of Population Health, University of Liverpool, Liverpool, United Kingdom.

Abstract:

Background: Research on the outcomes of veterinary treatments in dogs, cats and horses has important benefits for these animals and their owners. However, this information is not always available and the evidence base is often lower-quality than in human medicine. To identify ways to improve the generation of evidence, we investigated the views of veterinary professionals about research involving companion animal patients and their owners.

Methods and Findings: This qualitative study used semi-structured interviews with veterinary surgeons and registered veterinary nurses working in UK companion animal clinical practice. Interviews were conducted with 20 veterinary professionals from different clinical settings including both those with and without previous experience of research involving animals. Analyses revealed that veterinary professionals valued evidence-based information to help them make informed decisions about treatment with owners. However, there was often not enough available evidence. Veterinary professionals were willing to help produce this research evidence. However, lack of time and resources were key challenges and in addition, they did not always feel they had the necessary research skills, experience and support. Some participants also found it difficult to discuss participation in research with owners of their veterinary patients. They also had concerns about the amount and type of extra information they would need to give to owners. Veterinary professionals also faced a dilemma as their key role is to protect the welfare of animals that they treat, yet felt that there was the potential for some animals assigned to receive a specific treatment as part of a research study to be disadvantaged.

Conclusions: Companion animal research has important benefits for veterinary patients, their owners and for veterinary professionals. More funding, improved research training, resources, support networks and changes to current regulations are needed. Improved

evidence would assist veterinary professionals and owners when making informed decisions around veterinary care.

Introduction

Evidence-based veterinary medicine (EBVM) began to emerge as a concept within the veterinary literature in the late 1990's [1, 2, 3, 4], following the new paradigm of Evidence-Based Medicine (EBM) that had emerged within human medical practice a decade earlier [5]. EBVM applies EBM principles, classically defined as "the conscientious, explicit and judicious use of current best evidence in making decisions about the care of individual patients" [6], within a veterinary professional context considering veterinary patient needs and client preferences within the process of making a clinical decision [7, 8]. EBVM has been promoted globally within the veterinary profession, is incorporated within undergraduate and postgraduate training programmes and is embedded within professional regulatory requirements. At the same times there has been the development of resources and networks including evidence syntheses, evidence-based clinical guidelines and improved access to published research [9].

At the same time,

In human healthcare, EBM is promoted and facilitated through a variety of professional and academic networks e.g. British Medical Journal Best Practice support tools [10]. Regularly updated, high quality, accessible synthesised evidence generated by organisations such as the Cochrane Collaboration are also available to address important, current questions around health and care decision-making [11]. This requires availability of high quality, unbiased, primary evidence involving engagement of medical and other healthcare professionals and patients with research studies. These are underpinned by well-established ethics frameworks governing studies involving human participants. However, concerns around clinical research involving vulnerable patient populations, such as children, who cannot usually consent for themselves adds to the complexity of undertaking patient-based research, potentially

excluding those populations from clinical research and evidence production and the associated benefits of EBM [12].

The latter complexities have important parallels with research involving veterinary patients where owners of animals are required to provide consent on their behalf. Previously identified barriers to the use of EBVM within veterinary clinical practice [13,14,15] include a lack of available high quality primary evidence based on research conducted in veterinary patients in clinical settings. However, exclusion of veterinary clinical patients from high-quality primary research also excludes this population from the benefits of EBVM. Whilst research involving experimental laboratory animals is tightly regulated and relatively consistent across countries, currently, there is no internationally agreed framework for the involvement of animals in receipt of veterinary care with regard to their participation in research [16]. Ethical regulations are set at a national or regional level and with variations in ethics, regulations and professional body frameworks between countries. Within the UK, veterinary clinical research (VCR) is defined by the RCVS as routine procedures undertaken for the benefit of the animal/s, with the concurrent intention to generate new knowledge that benefits animals and requires ethical approval [17]. This is different to the RCVS definition of routine veterinary practice (RVP) where procedures and techniques are performed on animals by registered veterinary surgeons (or registered veterinary nurses under their direction) in the course of their normal professional duties (i.e. not for the purpose of generating new knowledge). Where a procedure or treatment is not considered to be RVP, additional regulatory approval is required [17].

Medical professionals are frequently involved with patient-based research with increased calls for research to become normalised as core business within the NHS in the UK [18]. However, veterinary patient-based research is less common despite its importance to veterinary professionals and animal owners alike [19]. Identification of factors that motivate or act as barriers to engagement of veterinary professionals with EBVM has been the subject of other studies [15], yet limited research has been conducted around the role of veterinary professionals in generation of primary evidence as part of EBVM. The aim of this qualitative study was to explore UK veterinary professionals' views around their own contribution to primary evidence generation through research involving client-owned companion animal veterinary patients (dogs, cats, and horses), and their assessment and application of existing research to inform clinical decision-making.

Methods

Ethics statement

The study was approved by the University of Liverpool Veterinary Research Ethics Committee (VREC1193). Informed consent was obtained from all study participants. Recruitment of participants took place between 02 June 2022 – 23 February 2023.

Study design

This qualitative study used interviews of UK-based veterinary professionals (veterinary surgeons and registered veterinary nurses) to provide in-depth exploration of participant's

views and experiences of research involving client-owned companion animals (defined for the purpose of this study as dogs, cats and horses) and contribution to, and use of, EBVM.

Key areas that we sought to explore included: veterinary professionals' prior experience of and willingness to participate in patient-based companion animal research including interventional studies such as clinical trials; opinions around commercial (pharmaceutical industry) vs academic (charity or veterinary sector funded) research; practical challenges in recruitment of patients and study conduct including data collection. A semi-structured interview script (Appendix 1) and vignettes of 3 clinical studies (Table 1) was developed and refined following feedback from veterinary professional colleagues. The vignettes placed the research activity in different types of research context; an academic research study investigating two medical therapies and surgical procedures considered to be routine veterinary practice (RVP according to RCVS guidelines) but to which a patient would be randomly allocated to (therefore defined as clinical veterinary research [CVR]) and a commercial (pharmaceutical industry funded) clinical trial investigating a novel therapy in client-owned veterinary patients.

Table 1: Vignettes used during the interviews to stimulate discussion around a semi-structured interview guide.

SCENARIO	STUDY DESCRIPTOR	SMALL ANIMAL EXAMPLE	EQUINE EXAMPLE
A	Randomised study comparing two routinely	Your practice has been asked to take part in a study comparing two routinely used medications (medication A & medication B)	Your practice has been asked to take part in a study comparing two routinely used medications (medication A & medication B)

	used medications	used as painkillers following orthopaedic surgery in dogs. Dogs would be randomly allocated to receive either medication A or medication B. You (or the vets in your practice) usually use medication A and think it works well but have not used medication B.	used to medicate joints for management of arthritis. Horses would be randomly allocated to receive either medication A or medication B. You (or the vets in your practice) usually use medication A and think it works well but have not used medication B.
B	Randomised study comparing two standard surgical techniques	Your practice has been asked to participate in a study comparing two commonly used surgical techniques for performing a bitch spay (technique A and modified technique B). Dogs would be randomly allocated to undergo either technique A or technique B. Within the practice vets perform both techniques but some vets prefer technique A and others technique B	Your practice has been asked to participate in a study comparing two commonly used surgical techniques for performing equine castration (technique A and modified technique B). Horses would be randomly allocated to undergo either technique A or technique B. Within the practice vets perform both techniques but some vets prefer technique A and others technique B.
C	Commercial drug trial	Your practice is asked to participate in a commercial drug trial looking at a new drug in cats diagnosed with Feline	Your practice is asked to participate in a commercial drug trial looking at a new injectable medication to treat Cushing’s disease (PPID). The

		Infectious Peritonitis (FIP). The product has been used in another country and the company are looking to get it licenced in the UK by conducting a clinical trial assessing safety and effectiveness of the drug. All drug and associated costs are covered by the drug company.	product has been used in another country and the company are looking to get it licenced in the UK by conducting a clinical trial assessing safety and effectiveness of the drug. All drug and associated costs are covered by the drug company.

Participant recruitment

Veterinary professionals (veterinary surgeons and registered veterinary nurses) working in, or with experience of, UK companion animal (small animal and equine) clinical practice and who were willing to be interviewed were recruited. Participants were initially recruited through advertisements on veterinary professional social media groups and platforms, and by snowball sampling. Initially, any participant who applied to take part was accepted in the study. As recruitment progressed, sampling was strategic to ensure a diversity of participants across the sector and including those without and with different types of prior research experience.

Interviews

Semi-structured interviews were conducted by TF (non-veterinary professional) using video-conferencing software (Zoom.com). Interviews were based upon the semi-structured interview script already described but were adapted to the individual's experiences. Video interviews were audio-recorded and professionally transcribed. Any identifiable data, such as the names of participants, veterinary practices, or involvement in specific research studies were anonymised, and the cleaned transcripts were then uploaded into NVivo (2020) for analysis.

Analysis

Analysis was based on a constructivist Grounded Theory as described by Charmaz [20], with a social constructionist epistemology[21]. Data were collected and analysed concurrently, meaning that initial analysis informed later interviewing and analysis. Initially, open-coding was performed, with TF reading each transcript and writing down impressions and initial thoughts. Secondly, more targeted coding was used to 'label' units of text which were of particular interest; for example, text that showed assumptions, values, actions, or language use. As more data were combined into the dataset, codes were continually refined, clarified, and re-named in order to incorporate the information within them, and to represent the ideas being expressed by participants. 'Memos', or notes regarding broader interpretation, were kept on a virtual whiteboard throughout this process. As the research progressed, the memos and codes were organised in order to create overriding themes which allowed the researcher to understand the views and experiences shared by interviewees.

TF also used memos to reflect on her positionality of the study; for example, her own prior experience as a participant in in several clinical research studies, decision-making and evidence-seeking around maintaining health of her own companion animals, and reflection on

prior discussions with veterinary colleagues around research. In line with the grounded theory approach, in the initial phases of analysis no particular lens or theory was applied so as not to limit or impact the coding process. As the analysis phases became more theoretical towards the very end of the process, existing theories and approaches which offered additional perspectives on the data were considered by TF in conjunction with EP and DA. At this later point of analysis, Zinn's body of work on perspectives on risk (Zinn, 2016b, 2016a) was identified as reflecting issues discussed by participants with regards to their clinical decision making, and thus provided a theoretical lens which added further insight to the analysis.

Results

Participants

A total of 20 participants including 15 veterinary surgeons (VS) and 5 registered veterinary nurses (RVN) were recruited from across the companion animal veterinary sector. In-depth interviews were conducted online for each except one participant who chose to provide detailed written answers to the questions via email. Study participant details including species, veterinary professional role and veterinary sector worked in are shown in Table 2. To preserve participant anonymity, details of participants' prior research experience are not individually reported. A total of 5 participants had no prior research experience (3=VS, 2=RVN), 4 had laboratory animal research experience but no clinical research experience (1=VS), had or were currently undertaking a formal postgraduate research (PhD) qualification but had no clinical research experience (3=VS), 8 (6=VS, 2=RVN) had some experience of clinical research (e.g. as a collaborator or principal investigator or an

Figures below 10 should be written in words

observational study) and 3 were experienced in clinical research and conduct of interventional studies either as a research nurse (1=RVN) or principal investigator (2=VS).

Table 2: Features of veterinary professionals who participated in interviews.

Companion animal species worked with	Veterinary professional type	Veterinary sector worked in
**Small animal**	Head veterinary surgeon	Primary care – University teaching practice
**Small animal**	Veterinary surgeon	Primary care – University teaching practice
**Small animal**	Veterinary surgeon	Primary care – private veterinary practice
**Small animal**	Veterinary surgeon	Referral hospital - private
**Equine**	Registered Veterinary nurse	Equine hospital (primary care and referral)
**Small animal**	Registered Veterinary nurse	Research nurse - Referral hospital
**Small animal**	Veterinary surgeon	Primary care – private veterinary practice
**Small animal**	Registered Veterinary nurse	Primary care – private veterinary practice
**Small animal**	Registered Veterinary nurse	Research nurse – University referral hospital
**Small animal**	Veterinary surgeon	Referral hospital & research - University
**Equine**	Veterinary surgeon	Self-employed

**Small animal**	Veterinary surgeon	Primary care – private veterinary practice
**Equine**	Veterinary surgeon	Primary care – private practice
**Small animal /mixed practice**	Veterinary surgeon (practice partner)	Primary care – private practice
**Small animal**	Veterinary surgeon	Referral hospital
**Equine**	Veterinary surgeon	Referral hospital - University
**Equine**	Veterinary surgeon	Pharmaceutical company
**Equine / mixed practice**	Veterinary surgeon	Primary care – private practice
**Equine**	Registered Veterinary nurse	Primary care – private practice
**Small animal**	Veterinary surgeon	Primary care

Results

In this study the veterinary professionals interviewed described working in a culture which they perceived to value ongoing learning and a sense of "professional curiosity", yet, paradoxically, the practicalities of working as a veterinary professional hampered their ability to keep up to date with new evidence, or participate in evidence generation. Use of EBVM principles was viewed by participants as a fundamental part of their veterinary practice, involving questioning their own current practice and seeking new information to provide optimal treatment for the benefit of their veterinary patients and owners. Clinic-based research was perceived as being integrally tied in with EBVM given that it involves

questioning dogma and producing new evidence for continually improved practice. However, the structure of the veterinary profession was viewed as problematic by study participants, with veterinary professionals feeling overworked and lacking time to undertake key clinical tasks, making it difficult to actively engage in other activities such as research. The structure was at odds with the values of the veterinary culture, because it promoted "status quo" treatments and interventions, hampering participants' ability to be curious and engage in ongoing learning or evidence generation. In Fig 1 the interplay between the individual, culture, structure and EBVM is illustrated.

Fig 1: Model showing the interplay between factors found in this study

Contributing to the evidence base: veterinary professionals' views on client-owned companion animal research

Most participants were familiar with the concept of CVR and held a favourable view of running research studies within a clinical setting. All participants agreed that pragmatic research questions which answered every day clinical scenarios should be prioritised, and that patient-based research would provide genuinely useful, robust primary evidence. Engaging in research studies and new evidence synthesis was compatible with a sense of "*professional curiosity*"(P13), in which veterinary professionals reflected on their practice and sought new information. Research was seen as potentially adding to the collective evidence base for day-to-day veterinary practice:

> P2: *at the end of the day vets are scientists*

P15: *I think if we are to make progress and not just reliving Groundhog Day style the same mistakes of all our predecessors we are going to need to engage with clinical research, and in an environment where it's relevant…..*

Additionally, while in theory participants were positive about taking part in clinic-based studies, the case study scenarios provided insight into some more specific areas of contention. Every participant discussed the need to minimise potential harms to veterinary patients participating in research studies. For some participants these potential risks needed to be reduced so far as to make the experience of an animal enrolled in a research study indistinguishable from an animal undergoing standard veterinary care. For example, comparing two known and licensed analgesics was generally considered ethically permissible, but studies involving a medication with unknown or limited evidence of efficacy raised concerns. Responses to the scenarios exposed a tension between the veterinary professional acting as a provider of care for the individual animal, and a researcher who was using an animal to generate clinical evidence, and all participants described "animal welfare" as their first priority. As a result, studies that might expose animals to any level of negative experience were considered problematic and potentially unethical:

P1: *I think if you were doing a drug A versus drug B I'd need to know that drug B works. I know the whole point of testing is to test which one is superior, but I'd need to know that the drug had pain relief, and was proven for pain relief, on its own. I know it probably would have been, through the licensing process, anyway. But I'd need to know that I'm not, sort of, permitting half my dogs to be in pain*

P16*: I won't give a placebo if I don't feel that it's in the animal's best interest and I'm a very clinical clinical researcher. I have always done, I've observed, collated, asked questions and you know performed interventions that I feel are in the animal's best*

interests and compared with standard treatments. But I've not withheld treatment for the sake of research and I would never do that

Placebo-controlled studies were generally considered unethical by participants in this study, due to the need to withhold treatment from the control group. However, randomised trials comparing known interventions were perceived as acceptable because both groups of patients would receive treatment.

Randomised studies were sometimes contentious for participants, because of the need to discuss the lack of evidence-base for an intervention with clients. Some participants described that in clinical circumstances they would simply give a client a medication or advise a particular intervention, and the client would trust that their animal had been given an appropriate treatment, usually without further discussion about the available evidence for the treatment. However, in a research scenario, the lack of evidence for the intervention and control would be described to the client as part of the consent process, creating an unusual client-veterinarian dynamic:

P14*: most owners, they go home with the dog's painkillers, you know, now they wouldn't know if it was A. or it was B. They would just assume you are going to send it home with pain relief. You're then saying to them right I'm going to give them painkiller A. or I'm going to give the dog painkiller B. they are going to say well what's the difference, you know "I don't know".*

This was termed "revealing uncertainty" in the coding, and incorporated ideas around managing animal welfare with the unknowns which were described overtly in the research scenario, yet remained hidden in standard care, with the client more frequently placing their trust in the expertise of the vet and the recommended treatment. Revealing uncertainty to clients as part of the research procedure flouted the expected norms and trust between

professional and client. This unfamiliar dynamic could be perceived to undermine vets' credibility to some extent, and also place an additional burden of decision-making on clients, who may have to weigh up evidence themselves.

However, participants discussed instances within veterinary practice during which uncertainty was present, yet without the same level of concern. For example, newly licensed drugs, off-label usage, or veterinary "specials" (extemporaneous preparations of a product without marketing authority) as part of clinical care did not usually concern participants in the same way as a patient-based research study. In this situation, veterinary surgeons described that they would inform the client that the product was off-label or a "special", but suggested that the depth of discussion was different to a veterinary patient in a research study, in which all aspects around potential uncertainty were required to be explicitly stated to the owner of that animal. In this way, the relationship and dynamic of trust between client and professional was maintained, despite uncertainty.

As a result of the need to reveal uncertainty around interventions in patient-based research and the need to protect animals from potential harm, most participants considered Scenario 1 (a randomised trial on two licensed drugs) to be acceptable only when there was adequate available data to support the efficacy of both drugs. Additionally, a "rescue protocol" (a pre-agreed plan for analgesia if the study drug is not working) was also viewed as necessary by participants with prior experience of conducting these types of studies. Participants had an additional concern about Scenario 2 (a randomised study comparing two routine surgical techniques for a procedure) because of the potential for a veterinary surgeons lack of familiarity with the procedure to add a layer of risk for the patient:

> Participant 9: *I would worry that the vets would have, if one vet was given technique B, but they're used to doing technique A, then I would maybe have a little bit of*

reservation about how skilled the vet is going to be in doing that technique…Would they then struggle to do it in this new technique that they're not quite confident with, which then could have an impact on the surgery time.

Contrastingly, Scenario 3 involved a pharmaceutical company funded clinical trial investigating a novel therapy for management of a chronic condition, feline infectious peritonitis (FIP), in the small animal context, and pars-pituitary intermediary dysfunction (PPID) in the equine context. In this instance, respondents were generally comfortable with offering the drug given the few available options for both conditions. In comparison to PPID, FIP is a usually fatal condition with no effective therapies previously available, and hence participants described that they would be comfortable trialling a new medication under this circumstance:

P8: *in the case of FIP we are often stuck as to what we are going to do. So, I would be quite happy in that sense to sort of say yes this is something we could try*

P2: *FIP essentially, for most cats is a death sentence. So, I think if there's any option of treatment of trialling something that could prolong life or effectively treat it then that's a good thing*

Across all three scenarios and differing professional and personal experiences, participants frequently imagined discussing informed consent with owners of eligible animals, in order to help them weigh up their own values and ethical views around the research study:

P14: *back 10, 15, 20 years, FIP was a death sentence so you'd say to an owner "your cat has got FIP. We've got two choices. I can put your cat to sleep* now or we can fill *it full of some steroids for a few weeks and then we will put it to sleep in a few weeks/months". Alternatively, "here is the new drug, don't worry about the cost because that's all being covered, now we can try it but your cat you know might have*

side effects, might have problems, it might die. But I've just told you your cat is going to die anyway, we can try it, it might work so we've got nothing to lose have we?"

The prospect of having a discussion around informed consent with an owner acted as a mechanism for participants to evaluate their own ethical views around the research study, in the context of specific client-animal dyads; for example, taking some responsibility for deciding whether a client and their animal would be suitable, or it was in their interest to be invited to take part. Informed consent was described as a process taken extremely seriously by all participants. Participants described multiple ways that they would ensure that clients maintained autonomy when making decisions around their animal being recruited onto a clinical trial. Examples given included simplifying language, assessing comprehension, asking the question about research participation at an appropriate time, and being honest about the potential risks and benefits of participating:

> P19: *if I am asking the client to consent, I try to keep my opinions out of it, and just give them the facts and let them kind of decide unless they asked my advice on it… if I was unhappy with the level of understanding with the owner, and I didn't think that they could do informed consent, then I would be reluctant*

> P1: *the only thing would be making sure that I'm not accidentally giving them false hope.*

Participation in studies place responsibility on the owner to make an ethical decision on behalf of their animal. In order to make this decision the veterinary professional is required to convey often complex concepts to the client to ensure that this decision can be made. However, participants described situations where clients had not listened during the informed consent process for standard clinical interventions such as companion animals undergoing routine neutering. This made participants question whether animal-owners could play their own part in the decision-making process effectively:

> P14: *the problem with B. is you know an owner is coming in, they are overwhelmed with all the information about the big technical surgery I am going to do today anyway.*

Barriers to participation in patient-based research

Prior to taking part in research studies, several participants described feeling that research was interesting yet intimidating potential component of veterinary work; several described that it was perceived as something done by people with specific attributes, skills and knowledge:

> P4: *I think a mental barrier at least in my head probably until working alongside people like, realising you didn't have to be super smart and academic to do that kind of stuff [research], actually it was a range of people.*

> P17: *I always thought it would be really interesting to do research in clinical practice and actually it shouldn't be that hard to do. You should be able to do it, it should be fine, but knowing where to start was always off-putting and I didn't know where to take the first step, how to do it*

Often "knowing where to start" was a significant barrier; for example, knowing how to design a study, find out about licenses required or how to submit an ethics application. This could be frustrating for some. For example P13 had research questions and had stored clinical samples as part of his routine work outside the UK, with the knowledge that they would be interesting to research, but did not have the knowledge, time or funds to take their research question further and had been unable to source collaborators:

> P13: *it* [research] *certainly is something that interests me. It's just very, very difficult to know how to get into it. As regards research I do very, very much have a professional curiosity about things and so do my colleagues, but how that translates into research is kind of difficult. We often don't know, we've some really good data and we often don't know how to use it.*

Other participants, too, described the difficulty in seeking collaborators who would help them to get involved in clinical research:

> P17: *I wish there was an easier forum that would probably have to go through a university of people being able to sign up to clinical research in practice. So, Professor whoever is running this study, do you want to be involved in it and having a way you can go and look at that and get involved, because necessarily coming up with the questions is quite hard. Coming up with that initial stage is quite hard, but I think if it was a case of presenting it to a client, getting a form filled out and then reporting the data, I think there would be a lot of vets who would be interested in doing that*

Conversely, however, participants who were confident leading patient-based research studies reported finding it difficult to engage veterinary professionals to take part in them. P15, for example, had prepared a research protocol with a pragmatic research question which they felt could be easily answered by performing a low-risk study in collaboration with other veterinary practices. They described how they worked with veterinary professionals within those clinics to ensure that they were an integral part of the research, designing easy to use data collection materials, and providing ongoing support. However, they found that clinical staff did not enrol animals into the study due partly to time constraints and the pressures of general practice:

> P15: [at the beginning] *they were brilliant, they were super supportive and there was a massive enthusiasm to get involved in this. All the centres took home all the kit and stuff*

and very, very positive feedback initially. But again, I think that people are very busy, there are lots of other stresses and they find excuses, or good reasons I'm sure not to enrol any particular patient and after a year you ask them how they're doing and they say "well not brilliantly".

As demonstrated in P15's example, a lack of time or concern about disrupting fast-paced veterinary clinical practice was the most-often cited reason for not participating in clinic-based research. This was followed by concern by some participants about recruiting eligible animals due to a lack of confidence in the about the ability or willingness of the owner of that animal to provide consent. Additionally, participants described feeling that clinic-based research was onerous and complex, and might also mean less time spent with their patients due to the need to spend more time on paperwork. Veterinary professionals who had experience of running patient-based research studies in clinical settings described the need to work collaboratively with veterinary professionals within the research team and with animal owners, in order to overcome these barriers by increasing confidence and a sense of ownership of the research project:

P16: *if you come to your team of residents, interns and staff and say "oh I've got this funding aren't I clever", you are just going to put their back up, and they are just going to see this as extra work…* [in a past project] *I had an administrator at the time,… I said to her "are you interested in helping me with you know looking after these cases?". She loved collating all the information and she worked as research assistant part time for me and off we went, and again working together as a team with a problem, going together to get the ethics, going together to get the funding that's how you bring in the team, you don't turn up with a project"*

As P16 reported, the manner in which a research project was presented influenced the willingness of others individuals to take part in patient-based research tasks outwith their daily professional responsibilities. However, as noted by P15, results were mixed, thus hampering their own efforts to perform ongoing research studies.

Stepping stones to engaging veterinary professionals with patient-based research

Several participants described how they used clinical audit as a means of getting other veterinary professionals to take an initial step into research. Clinical audit was viewed by some participants as a cross between clinical practice and research in a way that produced meaningful results, but without many of the methodological components such as research ethics approval, informed consent, or sample collection. Participants who became involved with research either did so by proxy; for example, helping take samples for a study being run by others, or through post-graduate training such as undertaking a professional Certificate, Master’s degree or doctoral training. This helped to build their confidence around their own research skills in a situation where time had been specifically allocated for this purpose.

Once engaged in research studies, participants reported a range of benefits, from improved team-working through to learning new skills, and seeing additional career opportunities. This was particularly emphasised by registered veterinary nurses, who found a new niche available to them; two of the participants were employed specifically to work on research studies:

> P9: *The career opportunities are massive … I don’t think I realised when I was in first opinion practice I thought it was kind of like veterinary nursing in a first opinion practice, in a referral centre, or maybe a rep and that was it. But actually, there is so*

much more that vet nurses can do lecturing, teaching, research, there's just so much more that I just didn't realise before I started this really

Participants who had prior experience of undertaking patient-based research also described how taking part in studies potentially offered enhanced opportunities to spend time with animal patients and their owners during study visits. However, those who had no prior research experience were concerned that doing so might limit their time spent with other non-participating clients and animals, due to demands of time required to complete the required research paperwork.

Participants also described the importance of making the research study as pleasant as possible for the veterinary professional, animal and its owner(s), by making study procedures as straightforward as possible and making sure the client felt that their contribution to the study and collective veterinary knowledge was appreciated. Generally, clients were said to respond favourably to being asked whether they might be willing to enrol their animal in a research study, even when the study requirements were quite onerous.

P4: *It was to our advantage that we were offering, at least with that study, a very high standard of veterinary care.. it probably was about £200 worth of veterinary care a year which, especially for a lot of the people in that socioeconomic sort of demographic and the part of [place] were the clinic is, that was a really big incentive, they loved to be able to provide something like that for their cat*

P10: *when I've explained, well, actually, if your dog participates now, when we publish all our findings, when this is out into practices, your dogs have had the opportunity to try and prevent other dogs from going years and years and years with having a lameness that hasn't been detected…. And that's I think, when they've been*

able to see the benefit of the research, in later years for dogs in the future. That's what's really sparked their interest. And they've all been really, really enthusiastic

Participants with experience of conducting veterinary clinical trials also described ways to potentially reduce some of the concerns that less-experienced researchers may have. This included the protocol for withdrawing clients' animals from the study, or using a rescue medication if needed.

> P5: *I know that within our randomised trials if, at any point, the owner of the horse has any concerns that horse will be removed and then you can unblind that case and you can then continue treatment accordingly. So, I guess the option is always there to take a step back and to take yourself out of that situation if you were not happy with it.… I equally don't feel that anybody within our industry would do something they felt would be detrimental to the horse*

In this way, concerns about maintaining optimal animal welfare during a patient-based research study were overcome, clients had reassurance of their autonomy over their animal's ongoing inclusion, and it provided reassurance to veterinary professionals who had concerns around potential ethical issues.

A culture of curiosity: use of evidence-based veterinary medicine (EBVM)

EBVM was constructed as a central component of veterinary professional culture, comprising decision-making based on evidence from clinically-relevant research studies. Participants described how they strived to maintain an up-to-date knowledge of evolving research findings. Use of skills in critical thinking to apply this knowledge to veterinary clinical practice was thus constructed as a central tenet of being a "good" veterinary professional. As

part of this willingness to learn and update their own professional practice, participants were encouraged to question dogma and traditional practices:

> P16*: I think there's a real push for every vet in practice to question even the basics, you know, should I be disinfecting the skin before I give an IV injection or is it better to be dry. You know, should I? And sometimes the answer is no and sometimes it not.*

> P13: *your mind is always thinking and turning over and you're asking yourself why or how, how could we do better. Should we be doing better? Are we making any difference or is it just time? Are we just going to do the same thing over and over and expect a different result?*

> *P12: so much of what we've done has been from, you know, James Herriot days and its only now that some of these things have been questioned. For instance we used to standardly neuter everything that walked through the door and now it's actually, we are questioning that*

The culture of the veterinary profession was described as valuing this mindset and a "*passion*" for continual improvement in patient care. A 'good' veterinary professional was viewed as being able to maximise health for animals in their care by being aware of the efficacy of possible treatment options and the potential for adverse effects. This enabled them to provide an informed presentation of potential options to animal owners, often to the point of being able to quantify risk for clients:

> *P5: I see it everyday, when people ring, particularly in the middle of a colic surgery and an owner says "right well what's the prognosis can you help me make my decision?" and actually I say the majority of time that that information in our environment comes from research and we are being able to put a quantitative figure on something to help people make a decision.*

Regular revisions of practice protocol due to evolving evidence were expected, and participants found it relatively easy to think of recent examples when the veterinary practices and hospitals they worked within had made changes based on new clinical evidence, for example, when practices altered their protocols around antimicrobial use. Individuals who did not show enthusiasm for keeping up to date were described as "*dinosaurs*" (P12), and considered to have "*lost their passion*" (P7), and were considered to be potentially poor veterinary practitioners because of their lack of awareness of veterinary advancements:

> *P1: I've definitely locumed at practices and worked at practices where I've not been senior and it's been on the opinion of the lead vet. And if they're someone who's very….traditional, stuck in their ways – I hate to say it, but old school boomer vets – it can be difficult to say "we should be doing X, Y, Z" because "Oh we've always done it this way, it's always been fine for me". It is then very personality dependent, but a lot of practices I've locumed at that's been the situation, I've then been offered permanent jobs and turned them down because I've sort of, self selected to be in the kind of teams I like to be in, which is open to new stuff, happy to change things around.*

Participants described how they saw it as part of their job to take personal responsibility for keeping up to date with new research findings via a range of methods including reading and critiquing research articles, reading news, discussing cases with colleagues, studying for postgraduate qualifications, attending conferences and seminars, attending continued professional development (CPD) events, and in-practice sessions organised by pharmaceutical companies, often referred to as "lunch and learn". These activities could be active (sought out by an interested individual) or passive (attending only mandatory CPD, or only in known areas of interest; going to lunch and learn sessions because they were convenient).

A passion for practising evidence-based veterinary medicine and a positive attitude to clinic-based research were considered to be integral to each other, because the mindset required to be a good veterinary surgeon was constructed in the same way as patient-based research, given both required the need to make use of a professionally curious mindset:

> *Participant 1: it's a funny one, trying to make people realise they do do research every day, it's just maybe not how they think of it. Not big drug trials with fancy professors, it's "look our cat castrates are looking poor in recovery, let's look at their pain scores and see if we can change something".*

> *P16: you asked me about research versus practice, but I think its categorising it as two different things. I think they're not two different things, I don't think you can be a good practitioner without asking questions.*

A structure of maintaining the status quo: difficulties practising EBVM

Despite the culture of ongoing learning, evaluating evidence and keeping up to date was not necessarily considered easy for participants attributed to structural issues within the veterinary industry. These predominantly related to the poor availability of an appropriate evidence-base for making evidence-informed decisions, such as inability to access journal articles, and limitations on professionals' time and energy, which meant that it was difficult for them to keep up to date with new information, or think about participating in clinical research. Combined, these issues contributed to participants' reliance on their previous experience and a "*paint by numbers*" (p15) approach to treating clients' animals, rather than maintaining a professionally curious mindset and conducting ongoing learning.

The context within which participants worked was textured by a shortage of veterinary professionals, a highly demanding workload, and clients who had increased expectations with

a willingness to resort to litigation to settle disputes. The task of keeping up to date with new research took a backseat behind the more immediate concerns of managing the day-to-day work context:

P12: *we are all stretched. And like my day yesterday was just horrific, constant, and sometimes you just don't feel like you're doing a good enough job for your patients and not caring for them as well as you could do just because you're literally being pulled from pillar to post and being hit like a dodgeball everywhere.*

P2*: I don't recall ever being at a practice where we routinely had journals clubs. It was attempted to be set up at the university practice when I first started there and it did last a little bit but unfortunately it was just, you're so time pressured, it wasn't able to be fitted in in the day. We tried to do it on the evening, but inevitably, by the time you finish at work in the practice its later anyway and then people want to go home.*

P17*: I think what lots of people would say, if you asked them why, what are you barriers to interpreting the evidence, they would say time. I don't have time to go and read papers, I don't have time to evaluate it, I don't have time to sit and think about it, I don't have time to drink a cup of tea!*

Additionally, most participants described feeling that, even with their best intention to practice EBVM, there was a lack of clinical evidence on which they could base their practice. Examples given included the frequency of veterinary research studies that had small sample sizes and thus low statistical power, or because studies were not considered applicable to a general practice setting:

P7: for most drug trials, the study numbers are poor aren't they…. most maybe like 40, 50 patients in a trial and although, you know, maybe the P Value says that it is

statistically relevant it still doesn't feel sometimes like you can completely just trust that, I don't know.

> P16: *You only have to look at Veterinary Evidence, the journal which looks at knowledge summaries and you know more than half of them go well there is not enough evidence to answer this question, there is just not enough evidence. A lot of the evidence is poor quality*

> P15: *I would love to work out those doors into* [conducting research in] *primary practice, because ivory tower work done in universities doesn't always apply to the standard treatment*

In addition, the structure of funding for patient-based research and research publication was viewed as potentially heavily biased. Participants reported being unable to trust that published information provided an honest reflection of the results of research studies, particularly if those results were generated or funded by organisations with a vested interest in the results, such as a pharmaceutical company. Keeping up to date with new studies therefore required not only reading the available evidence, but critically appraising the methods and funding models. Some respondents described that establishing the "truth" of a treatment was complex, time-consuming, and sometimes disempowering:

> P14: *I think there's a lot of information but trying to then narrow that down, or trying to filter it out, I think that often is the difficulty, you know that you can, especially with something as grey and non-black and white as medicine and biology, there can almost be, for every argument for something you can almost find something that's against it, for me to know in general practice if a particular study is flawed or not, or how good that study was, you know on something that I'm just reading online - I am not skilled*

enough to know how, 'cos there are studies, as you well know, there are studies and there are studies aren't there.

Contrastingly, many participants described trust in the licensing process, thus being reassured that a licensed product was appropriately safe and efficacious for use in clinical practice:

> P12: *From an ethical point of view having done the work I've done, I know that in order to get the licenses and permissions and the ethics approval then its gone through the various hoops to get thro*
>
> *ugh that*

> P19: *As long as it's proper authorised veterinary medicine, and proper trials I wouldn't be too concerned. But there's always a little bit of concern when you use a new thing.*

Licensing was one example of many, in which where participants described relying on trust (whether in a process, a brand name, or a type of industry e.g. academic research vs pharmaceutical), tradition, or experience to allow them to function in the busy and stressful veterinary companion animal clinical environment.

Discussion

While veterinary professionals prize EBVM and view professional curiosity around veterinary medicine as part of being a “good vet”, they reported that working as a veterinary professional meant that they had a lack of knowledge and time to read about or take part in research. To our knowledge, this is the first study to investigate veterinary cultures around knowledge production and application in client-owned companion animal patients. Our findings have identified potential approaches to improve generation of primary evidence and promote engagement of veterinary professionals in patient-based research.

The acceptance of EBVM principles and aspiration to utilise evidence-based practice as a responsible, modern veterinary professional is not just a “passion” as described by some participants, but an epistemological approach and belief system with roots in medical literature which prioritises the seeking of “truths” via science [22]. Participants described that VPs should have a sense of ‘professional curiosity’ in using knowledge produced through scientific research to try to quantify uncertainty in the management of clinical conditions or determine whether equipoise exists. However, they described that they were constrained by the pressures of service provision, and current pressures in the veterinary field pertaining to the cost of treatments, transparency of available options, and clear information around treatment outcomes[23]. Clinical research has an important role to play here in clarifying treatment efficacy and increasing the treatment options available, by adding to the knowledge base of clinical evidence. This information aligns with Zinn’s “rational” approaches to risk, in which positivist cognitive approaches to decision-making are viewed as more effective than “irrational” approaches, which include hope or faith, or “in-between strategies”, such as trust or intuition [24].

However, participants also understood that the evidence generated through veterinary patient-based research was constructed through complex, socially constructed processes where certainty was not always achievable [25]. Borrowing from Zinn's work applied to the veterinary clinical setting, participants valued research that armed them with logical answers to clinical questions, such as the best treatment option for a condition. Whilst they presented themselves as predominantly "rational" thinkers, the demands and structure of veterinary clinical practice resulted in numerous examples of "in-between" approaches to risk at play [24,26]. Practice was constructed as a colourful, fraught, messy reality of interaction between professional, client, and animal where rational approaches were problematic and, sometimes, impossible [27].

Webs of trust and other "inbetween" approaches to navigating clinical evidence and decision making were also identified in our data. Participants described the tensions around trusting clients to listen and understand options for treatment [28], sought science-based answers to clinical questions yet used intuition as part of "clinical skill" [29]; discussed trust in drug licensing and production procedures, and described emotions such as fear when trialling a new product. The uncertainty presented when conducting randomised studies in particular added additional layers of complexity. Participants overtly discussed moral tensions around evidence generation, including clinical equipoise and use of placebos [30,31].
Communication and trust between clients and veterinary professionals during clinical research has been researched, with a focus on informed consent for clinical procedures and protecting of veterinary professionals from alleged professional negligence [27, 28, 32, 33]. The extensive role of trust between client-professional in communication identified in this

study is important when considering veterinary professional perspectives on patient-based research.

Implicit uncertainties in practice are made overt during the research and informed consent process, by laying bare the holes in clinical knowledge which are often overlooked during veterinary consults [28, 34]. Initiation of discussion about a patient's potential participation in a research study and obtaining informed consent was therefore an important obstacle for some participants in the present study. This was further compounded by concerns around the additional paperwork and complexity of information [35] that veterinary professionals might be required to give to a client about a research study, particularly within a time-constrained clinical environment.

Based on the findings from the present study, there is a need for further research and training around communication between veterinary professionals and clients in the context of companion animal patient-based research. The development of evidence-based frameworks, similar to those used in medical research involving paediatric patients [36], could enhance veterinary professional explanation and client understanding, together with improved resources and networks on this specific aspect. Researchers and ethics committees must ensure that consent forms and associated information meet existing evidence-based healthcare readability guidelines [35, 37]. Engagement with owners of companion animals during the design of veterinary clinical research studies has important benefits [34]. Their involvement during the design of client information and consent forms may help to address concerns expressed by participants in the present study around the type of information presented to clients together with initiation of discussions and type of language used. This

process is important to ensure the process of informed consent has been met and to optimise engagement of veterinary professionals (and companion animal owners) with patient-based research.

The navigation of ethical practice and delineation of acceptable and unacceptable animal use in the very different context of animal research laboratories has been referred to as "ethical boundary work" [38]. Veterinary professionals in the present study also engaged in negotiating ethical boundaries, and described how these boundaries were coloured by the priorities of veterinary practice. In particular, participants discussed the tension between a veterinary professional's primary role to protect animal health and [17], the need to provide a reliable service to paying clients, and the practice of conducting research involving animal patients, in which subjects are put at some degree of risk or harm – even if those risks are relatively minor, to potentially benefit a species as a whole. These concepts could be seen to be ethically incongruous [16, 39], particularly given that some areas of clinical veterinary research, such as critical care research, have been criticised for poor practices, poor reporting, and potentially replicating studies unnecessarily [40]. Tensions between owner autonomy and veterinary professional primary duty of care towards animals were also expressed by some participants in the present study [28, 41]. Whilst primarily in favour of research involving client-owned companion animal patients, these "in-between" processes around negotiation of ethical boundaries led to participants describing relatively conservative views in relation to the specific research scenarios described in this study. Ongoing calls for an internationally agreed framework around regulation and ethical best practice in clinical veterinary research, considering the issues of autonomy and informed consent [16, 41] appears to be timely and important.

(Human) healthcare research offers important career opportunities for nurses and associated professionals, who are ideally placed to communicate with patients, collect data and to coordinate or run research studies[42]. The career benefits and personal satisfaction of being involved in patient-based research within a veterinary clinical setting was discussed by registered veterinary nurse participants in the present study. This approach offers multiple benefits in terms of career progression and retention together with cost and time efficiencies compared to an approach involving only, or predominantly, veterinary surgeons[42]. Improved opportunities for veterinary nurses to be involved in research with relevant training and support could provide an additional way to expand current primary research generation.

The limitations of traditional gold standard RCTs, which are highly regulated and can be prohibitively expensive and challenging to conduct, and value of primary evidence generated from well-designed, prospective observational studies utilising patient data are increasingly recognised within medical [43] and veterinary healthcare. Technological advancements in digital collection of patient data (human healthcare and veterinary) and use of 'big data' epidemiological approaches provide cost-effective ways of generating primary research evidence. This is particularly important in veterinary clinical settings where research funding remains extremely limited compared to human healthcare. Use of machine learning models provides cost and time efficiencies through interrogation of large volumes of unstructured, text-based veterinary electronic health records. These approaches are already contributing to generation of primary evidence from large veterinary companion animal patient populations [44,45] and is a rapidly expanding area of current veterinary clinical research. Nevertheless, RCTs, including pragmatic and newer adaptive and platform designs, remain the optimal way to obtain high-quality, unbiased primary evidence of efficacy of new therapies and treatment protocols in both human [46,47] and veterinary medicine [48]. Increased undergraduate and

postgraduate education around best-practice in the design and conduct of interventional studies including RCTs would be beneficial. Improved knowledge of key study features including suitable study power and awareness of stopping rules, and improved education to address ethical and welfare concerns discussed by some participants in the present study including clinical equipoise, use of placebos and monitoring of patient safety also appears to be required.

Limitations

This study investigated the views of veterinary professionals working in UK companion animal (small animal and equine veterinary) clinical settings. Therefore, the findings of the present study may not reflect the views and experiences of those working in other veterinary sectors or in other countries. Consistent with many qualitative studies, these findings are based on the results of in-depth interviews with a small number of veterinary professionals. We focussed specifically on companion animal patient-based research as this is very different from research involving production animal veterinary patients. Participants may also have been biased towards those with an interest in research and EBVM, although we tried to address this as best as possible through the recruitment process. However, our findings are broadly consistent with the findings from other research studies investigating EBVM in veterinary professionals and with qualitative studies that have investigated attitudes of medical and other healthcare professionals towards patient-based research. Additional qualitative research in this area is warranted including further in-depth study of veterinary professionals, companion animal owners and other stakeholders involved in companion animal healthcare.

Conclusion

Generation of high-quality primary evidence through engagement of veterinary professionals in patient-based research has important benefits for animals and their owners. This includes generation of improved evidence that veterinary professionals and companion animal owners can use during informed decision-making around options for veterinary care, including likely outcomes and associated costs. This is particularly relevant to the current Competition and Markets Authority review of UK veterinary services for household pets [23] in which concerns about the ability for pet owners to make informed decisions about veterinary care is part of the ongoing investigations. Continued improvement in knowledge and use of EBVM principles by UK veterinary professionals was evident [9, 49]. Application of EBVM was unquestioned as the goal for responsible, up to date veterinary professionals and as a means of reducing uncertainty by quantifying the effectiveness of veterinary treatments and other interventions. Participants unanimously agreed that the evidence base for veterinary medicine remains poor and the repeatedly identified need to generate the primary evidence through patient-based veterinary research [50] remains important. Participants reported willingness to participate in patient-based research and recognised the associated career opportunities and other benefits it provides. Lack of time, perceived poor research skills and confidence, limited resources and support and other structural issues within the veterinary profession were major barriers to engagement with research. Additional education, funding and capacity building is required, ulitising approaches used in global healthcare settings and paediatric medical research. This includes increased awareness of best practice in patient-based research, improved research skills and communication training, development of improved resources and communities of support and cost-efficient utilisation of digital patient data and increased use of research-trained registered veterinary nurses. Current ethical, legal and

regulatory issues relating to veterinary clinical research also need to be addressed at a national and international level.

Acknowledgements

professionals

We thank the veterinary professions who kindly agreed to participate in this study, and Dr Rebecca Smith for comments on an early version of the manuscript. Funding for this study was provided through the University of Liverpool Impact support fund.

Author contributions (credit taxonomy used)

Conceptualization: DA. Investigation: TF DA. Methodology: TF EP DA. Formal analysis: TF EP. Writing – original draft TF DA. Writing – review and editing DA, TF, EP

References

1. Bonnett, B., Evidence-based medicine: critical evaluation of new and existing therapies. Complementary and alternative veterinary medicine. St. Louis, MO: Mosby, 1998. pp.15-20.
2. Fogle, B., Evidence-based medicine. Vet Rec, 1998.143(23), p.643.
3. Malynicz G. Evidence-based medicine. The Veterinary Record. 1998 Nov 1;143(22):619-.
4. Jones WE., Evidence-based equine medicine. J Equine Vet Sci 2000 20(7):415.
5. Guyatt, G., Cairns, J., Churchill, D., Cook, D., Haynes, B., Hirsh, J., Irvine, J., Levine, M., Levine, M., Nishikawa, J. and Sackett, D., Evidence-based medicine: a new approach to teaching the practice of medicine. JAMA, 1992, 268(17), pp.2420-2425.
6. Sackett DL. Evidence-based medicine. InSeminars in perinatology 1997 Feb 1 (Vol. 21, No. 1, pp. 3-5). WB Saunders.
7. Holmes M, Cockcroft P. Evidence-based veterinary medicine 1. Why is it important and what skills are needed?. In Practice. 2004 Jan;26(1):28-33.
8. Schmidt, P. L., Evidence-Based Veterinary Medicine: Evolution , Revolution, or Repackaging of Veterinary Practice ? Vet Clin Sma, 2007 37, 409–417. https://doi.org/10.1016/j.cvsm.2007.01.001

9. Haddock, L., Baillie, S., Sellers, E. and Warman, S., Exploring the motivations, challenges, and barriers for implementing evidence-based veterinary medicine (EBVM) in general practice. Veterinary Evidence, 2023 8(1).
10. BMJ Best Practice [internet] BMJ. 2024. Available from: https://bestpractice.bmj.com
11. Cochrane.org [internet] Cochrane. 2024 available from: https://www.cochrane.org/evidence
12. Park, S.S. and Grayson, M.H., Clinical research: protection of the "vulnerable"?. Journal of Allergy and Clinical Immunology, 2008 121(5), pp.1103-1107.
13. Toews, L., The information infrastructure that supports evidence-based veterinary medicine: a comparison with human medicine. Journal of Veterinary Medical Education, 2011 38(2), pp.123-134.
14. Vandeweerd, J., Kirschvink, N., Clegg, P., Vandenput, S., Gustin, P., & Saegerman, C., Is evidence-based medicine so evident in veterinary research and practice ? History , obstacles and perspectives. The Veterinary Journal, 2012 191(1), 28–34. https://doi.org/10.1016/j.tvjl.2011.04.013
15. Haddock L, Baillie S, Sellers E, Warman S. Exploring the motivations, challenges, and barriers for implementing evidence-based veterinary medicine (EBVM) in general practice. Veterinary Evidence. 2023 Mar 16;8(1).
16. Ashall V., Morton D., Clutton E. A Declaration of Helsinki for animals. Vet Anaesth Analg. 2023 Jul;50(4):309-314. doi: 10.1016/j.vaa.2023.03.005. Epub 2023 Apr 5. PMID: 37183079.
17. Code of Professional Conduct for Veterinary Surgeons and Supporting Guidance [internet] Royal College of Veterinary Surgeons. 2019 Available from: www.rcvs.org.uk/setting-standards/advice-and-guidance/code-of-professional-conduct-for-veterinary-surgeons/pdf/
18. Arasaradnam R., Clarke S., Van't Hoff W., Chappell L. Making research everybody's business: a position statement of the Royal College of Physicians and the National Institute of Health and Care Research. Clin Med (Lond). 2023 102-105. doi: 10.7861/clinmed.2022-0482.
19. Hauser, S., & Jackson, E. L., A Survey of the Non-clinical Benefits of EBVM. Veterinary Evidence, 2017 2(3). https://doi.org/10.18849/ve.v2i3.102
20. Charmaz, K. Constructing Grounded Theory. Sage. 2014
21. Berger, P., & Luckman, T. The Social Construction of Reality: a Treatise in the Sociology of Knowledge (7th ed.). Penguin Books. 1991
22. Solomon M. Just a paradigm: evidence-based medicine in epistemological context. European Journal for Philosophy of Science. 2011 Oct;1:451-66.
23. Competition and Markets Authority. Veterinary services for household pets. 2024. [online] https://www.gov.uk/cma-cases/veterinary-services-market-for-pets-review
24. Zinn, J. O., 'In-between' and other reasonable ways to deal with risk and uncertainty: A review article. Health, Risk & Societyisk & Socity, 2016a 18(7–8), 348–366. https://doi.org/10.1080/13698575.2016.1269879
25. Janasoff, S., Technologies of humility. Nature, 450(November), 2007.
26. Zinn, J. O., The Sociology of Risk. In K. Korgen (Ed.), The Cambridge Handbook of Sociology. Cambridge Publishing Ltd. 2016b
27. Russell, E., Mossop, L., Forbes, E., & Oxtoby, C., Uncovering the 'messy details' of veterinary communication: An analysis of communication problems in cases of alleged professional negligence. Veterinary Record, 2022 190(3), no. https://doi.org/10.1002/vetr.1068
28. Ashall, V., Millar, K. M., & Hobson-West, P. Informed Consent in Veterinary Medicine: Ethical Implications for the Profession and the Animal 'Patient.' Food Ethics, 2018 1(3), 247–258. https://doi.org/10.1007/s41055-017-0016-2
29. Everitt, S. Clinical decision making in veterinary practice . PhD thesis , University of Nottingham, 2011.
30. Habing, G. G., & Kaneene, J. B., Stopping rules in veterinary randomised controlled trials. JAVMA, 2011 239(9), 1197–1199.

31. Miller FG. Clinical equipoise and risk–benefit assessment. Clinical Trials. 2012 Oct;9(5):621-7.
32. Gray C., Role of the consent form in UK veterinary practice. Vet Rec. 2020 Oct 17;187(8):318. doi: 10.1136/vr.105762. Epub 2020 Sep 11. PMID: 32917836; PMCID: PMC7606496.
33. Pun, J. K. H., An integrated review of the role of communication in veterinary clinical practice. BMC Veterinary Research, 2020 16(1), 1–14. https://doi.org/10.1186/s12917-020-02558-2
34. Fritz MC, Stanley BJ. Feline clinical trials: considerations for improving recruitment. Journal of Feline Medicine and Surgery. 2014 Sep;16(9):725-6.
35. Sobolewski, J., Bryan, J.N., Duval, D., O'Kell, A., Tate, D.J., Webb, T. and Moore, S., Readability of consent forms in veterinary clinical research. Journal of veterinary internal medicine, 2019 33(2), pp.350-355.
36. Roper, L., Lyttle, M.D., Gamble, C., Humphreys, A., Messahel, S., Lee, E.D., Noblet, J., Hickey, H., Rainford, N., Iyer, A. and Appleton, R., Seven-step framework to enhance practitioner explanations and parental understandings of research without prior consent in paediatric emergency and critical care trials. Emergency Medicine Journal, 2021 38(3), pp.198-204.
37. O'Sullivan, L., Sukumar, P., Crowley, R., McAuliffe, E. and Doran, P., Readability and understandability of clinical research patient information leaflets and consent forms in Ireland and the UK: a retrospective quantitative analysis. BMJ open, 2020 10(9), p.e037994.
38. Anderson, A., & Hobson-West, P. Animal research, ethical boundary-work, and the geographies of veterinary expertise. Transactions of the Institute of British Geographers, 2022a. September, 1–15. https://doi.org/10.1111/tran.12594
39. Taylor, P. and Meyer, R.E., Veterinary clinical research or experiments on pets. Veterinary Anaesthesia and Analgesia, 2023 50(5), pp.383-385.
40. Bara, M., Joffe, A.R. The ethical dimension in published animal research in critical care: the public face of science. *Crit Care* **18**, R15 (2014). https://doi.org/10.1186/cc13694
41. Hiestand, K.M., The autonomy principle in companion veterinary medicine: A critique. 2022. Frontiers in Veterinary Science, 9, p.953925.
42. Edwards, N., Webber, J., Mill, J., Kahwa, E. and Roelofs, S., 2009. Building capacity for nurse-led research. International nursing review, 56(1), pp.88-94.
43. Frieden T. R., Evidence for Health Decision Making – Beyond Randomized, Controlled Trials N Engl J Med 2017 377; 5
44. Burton, J., Farrell, S., Mäntylä Noble, P. J., & Al Moubayed, N., Explainable text-tabular models for predicting mortality risk in companion animals. Scientific Reports, 2024 14(1), 14217.
45. Singleton, D.A., Pinchbeck, G.L., Radford, A.D., Arsevska, E., Dawson, S., Jones, P.H., Noble, P.J.M., Williams, N.J. and Sánchez-Vizcaíno, F., Factors associated with prescription of antimicrobial drugs for dogs and cats, United Kingdom, 2014–2016. Emerging Infectious Diseases, 2020 26(8), p.1778.
46. Bhatt, Deepak L., and Cyrus Mehta. "Adaptive designs for clinical trials." New England Journal of Medicine 2016 375.1: 65-74.
47. Joshi, G. P., Alexander, J. C., & Kehlet, H., Large pragmatic randomised controlled trials in peri-operative decision making: are they really the gold standard?. Anaesthesia, 2018 73(7), 799-803.doi:10.111/anae.14238
48. Sargeant JM, Brennan ML, O'Connor AM. Levels of Evidence, Quality Assessment, and Risk of Bias: Evaluating the Internal Validity of Primary Research. Front Vet Sci. 2022 Jul 12;9:960957. doi: 10.3389/fvets.2022.960957.
49. Dean R., Heneghan C., Do we need an evidence manifesto? Vet Rec. 2019 Jul 13;185(2):58-59. doi: 10.1136/vr.l4653. PMID: 31296726.

50. Lanyon L., Evidence-based veterinary medicine: a clear and present challenge. Vet Record. 2014 Feb 15;174(7):173-5. doi: 10.1136/vr.f7519. PMID: 24526538.

Figure 1

Click here to access/download;Figure;image 1.tif

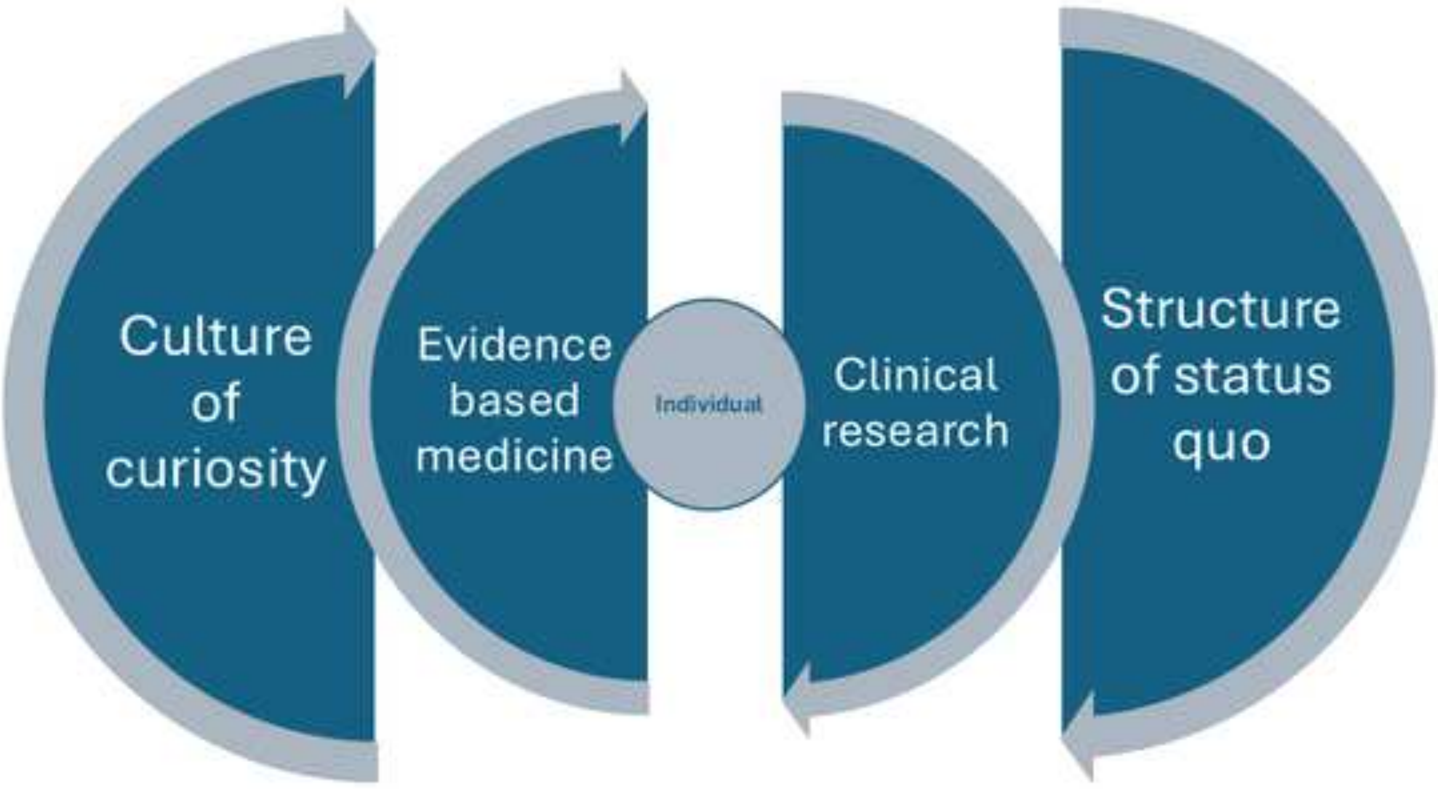

Click here to access/download
Supporting Information
Appendix 1 - Semi structured interview guide.docx
